# 515. The Impact of Prolonged Steroid Use on Critically Ill COVID-19 Patients: A Retrospective Cohort Study

**DOI:** 10.1093/ofid/ofad500.584

**Published:** 2023-11-27

**Authors:** Jaijun Han, Seongman Bae, Jiwon Jung, Min Jae Kim, Yong Pil Chong, Sang-Ho Choi, Sang-Oh Lee, Yang Soo Kim, Eui Jin Chang, Sung-Han Kim

**Affiliations:** Asan Medical Center, Seoul, Seoul-t'ukpyolsi, Republic of Korea; Asan Meidical Center, Songpa-gu, Seoul-t'ukpyolsi, Republic of Korea; Asan Medical Center, Seoul, Seoul-t'ukpyolsi, Republic of Korea; Asan Medical Center, Seoul, Seoul-t'ukpyolsi, Republic of Korea; Asan Medical Center, Seoul, Seoul-t'ukpyolsi, Republic of Korea; Asan Medical Center, Seoul, Seoul-t'ukpyolsi, Republic of Korea; Asan Medical Center, Seoul, Seoul-t'ukpyolsi, Republic of Korea; Asan Medical Center, Seoul, Seoul-t'ukpyolsi, Republic of Korea; Department of Internal Medicine, Asan Medical Center, Seoul, Korea, Seoul, Seoul-t'ukpyolsi, Republic of Korea; Asan medical center, Seoul, Seoul-t'ukpyolsi, Republic of Korea

## Abstract

**Background:**

Dexamethasone was found to decrease mortality in COVID-19 patients needing oxygen support. However, there is no clear guidance on the duration of steroid use after 10 days. This study aims to compare clinical outcomes of critical COVID-19 between patients abruptly discontinuing steroids within 10 days and those tapering off beyond 10 days.

**Methods:**

This retrospective cohort study included adult COVID-19 patients with a severity score of 6-9 on the World Health Organization-Clinical Progression Scale (WHO-CPS). Patients were divided into two groups based on the duration of maintaining steroids and compared for clinical outcomes such as incidences of rebound pneumonia and infectious complications, 28-day and in-hospital mortality, and readmission or revisit to the emergency room within three months after discharge. The study used a one-to-one propensity score matching analysis to eliminate potential confounding factors.

**Results:**

There were 121 patients who received dexamethasone for up to ten days and discontinue it abruptly and 139 patients who tapered off steroids after taking dexamethasone for up to ten days. After propensity score matching, 58 patients were included in each group and there was no significant difference in baseline characteristics between both groups. Duration of hospitalization was significantly longer in the steroid-tapering group (median 20.0 days, 95% confidence interval (CI) 13.0-29.0 days) than the group with abrupt discontinuation of dexamethasone (13.0, 95% CI 10.0-17.0) (*p*< 0.001). Also, there were six cases of rebound pneumonia in each group and 28-day mortality was 10.3% in both groups. Analyses after propensity score matching showed that there was no significant difference in 28-day and in-hospital mortality, the incidence of rebound pneumonia, infectious complications, and readmission or revisit to the emergency room between both groups.

Clinical characteristics of the study patients before and after propensity score matching

**Figure:**
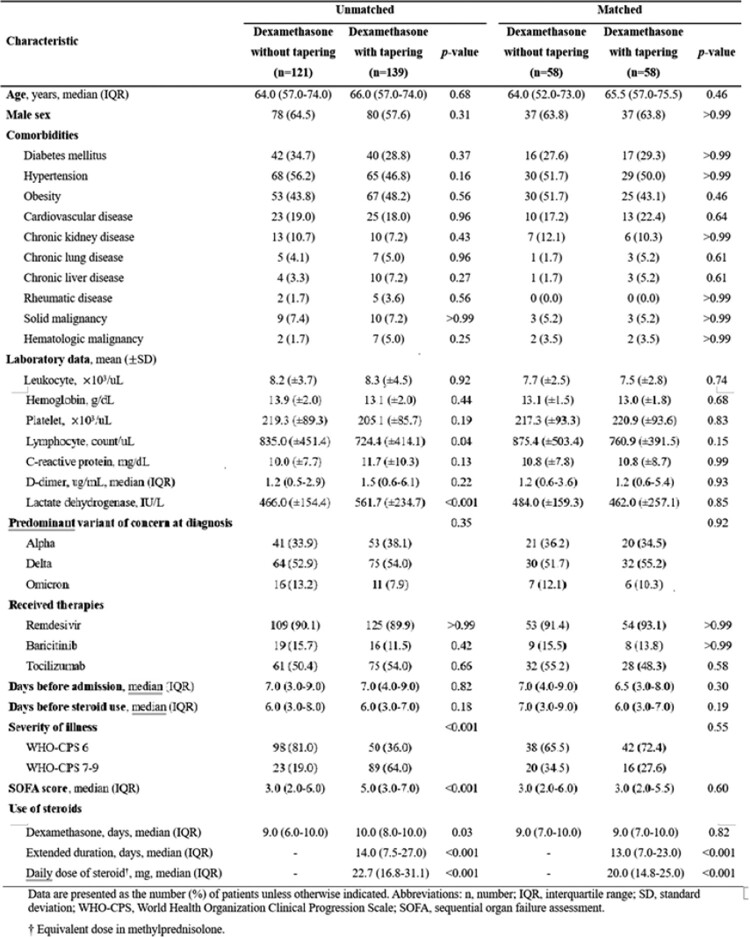

Clinical outcomes for the study patients before and after propensity score matching

**Figure:**
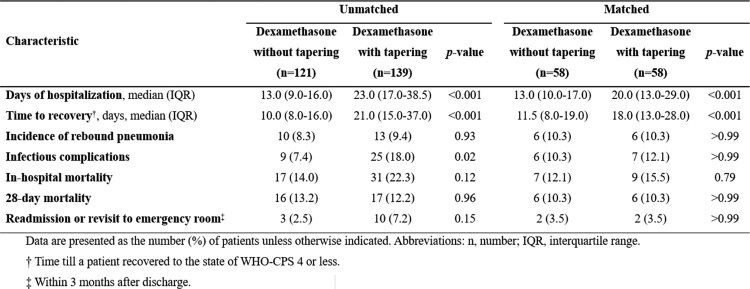

Survival analysis of 28-day mortality according to steroid tapering in critical COVID-19 patients

**Figure:**
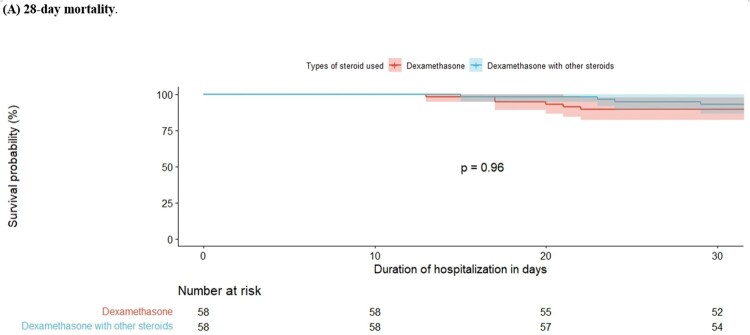

**Conclusion:**

Tapering and prolonged use of steroids after administering dexamethasone for up to ten days did not significantly improve the 28-day and in-hospital mortality, the incidence of rebound pneumonia, and readmission or revisit to the emergency room in critical COVID-19 patients with a severity score of 6-9 on the WHO-CPS.

Survival analysis of in-hospital mortality according to steroid tapering in critical COVID-19 patients

**Figure:**
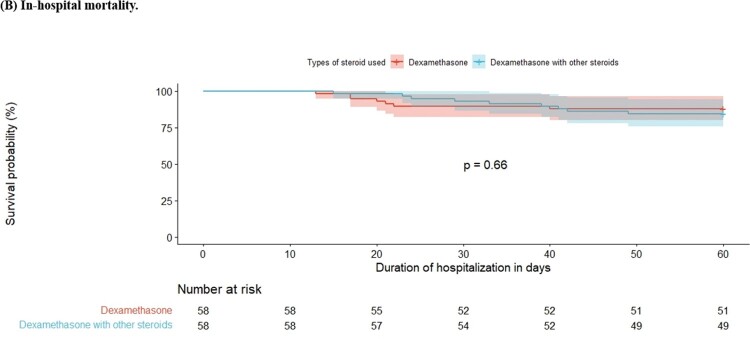

**Disclosures:**

**All Authors**: No reported disclosures

